# Correction: Parys et al. Inner Ear Pharmacotherapy for Residual Hearing Preservation in Cochlear Implant Surgery: A Systematic Review. *Biomolecules* 2022, *12*, 529

**DOI:** 10.3390/biom15060787

**Published:** 2025-05-29

**Authors:** Quentin-Alexandre Parys, Pauline Van Bulck, Elke Loos, Nicolas Verhaert

**Affiliations:** 1 Department of Otorhinolaryngology-Head and Neck Surgery, University Hospitals Leuven, 3000 Leuven, Belgium; quentin-alexandre.parys@uzleuven.be (Q.-A.P.); pauline.vanbulck@uzleuven.be (P.V.B.); elke.loos@kuleuven.be (E.L.); 2 Department of Neurosciences, Research Group Experimental Oto-Rhino-Laryngology (ExpORL), KU Leuven, University of Leuven, 3000 Leuven, Belgium

There was an error in the original publication [1]. The manuscript stated that the review protocol was successfully registered in PROSPERO.

A correction has been made to Section 2.1. Search Strategy & Study Selection, where one sentence referring to the PROSPERO registration was removed. The authors state that the scientific conclusions are unaffected. This correction was approved by the Academic Editor. The original publication has also been updated.
