# Supplementary material for: Inner Ear Pharmacotherapy for Residual Hearing Preservation in Cochlear Implant Surgery: A Systematic Review
Source: Biomolecules. 2022 Mar 31;12(4):529. doi: 10.3390/biom12040529 (PMC9032072; doi:10.3390/biom12040529)
Supplement: Supplementary file 1 [file biomolecules-12-00529-s001.zip › Material S1. Search Strategy.pdf]

Material S1: Search Strategy (as used for PubMed)

("Cochlear Implants"[Mesh] OR "Cochlear Implantation"[Mesh] OR (cochlea*[tiab] AND implant*[tiab]))

AND

(fibrosis[tiab] OR impedance[tiab] OR (auditory[tiab] AND brainstem[tiab] AND response*[tiab]) OR ABR[tiab] OR (hearing [tiab] AND preservation[tiab]) OR ((foreign[tiab] AND body[tiab]) AND (reaction[tiab] OR response[tiab])) OR (inner[tiab] AND ear[tiab] AND trauma[tiab]) OR inflammation[tiab] OR (inflammatory[tiab] AND (response[tiab] OR reaction[tiab])))
